# Exploring the Feasibility of Calculating Expected pCO2 From Venous Blood Gas Samples Alone in Intensive Care Patients

**DOI:** 10.7759/cureus.42944

**Published:** 2023-08-04

**Authors:** Semin Turhan, Duygu Tutan, Yeliz Şahiner

**Affiliations:** 1 Department of Anesthesiology and Reanimation, Hitit University Erol Olçok Education and Research Hospital, Çorum, TUR; 2 Department of Internal Medicine, Hitit University Erol Olçok Training and Research Hospital, Çorum, TUR

**Keywords:** acid-base disorder, winter's formula, partial carbondioxide, venous blood gas, bicarbonate

## Abstract

Introduction

This study highlights the significance of assessing acid-base balance and gas exchange in intensive care patients. The research investigates the applicability of using the "expected (pCO_2_ = HCO_3_ + 15)" formula, derived from venous blood gas samples, as an alternative to Winter's formula and practical formula. The study emphasizes the importance of identifying the primary acid-base abnormality accurately and efficiently for appropriate clinical intervention in critically ill patients.

Methods

This study included 400 adult patients admitted to the Anesthesia Clinic in the Third Stage Anesthesia and Reanimation Intensive Care Unit at Hitit University Erol Olçok Training and Research Hospital between April 2020 and July 2023. Blood gas samples were collected simultaneously from both arterial lines and venous catheters. Patients under 18 years, pregnant women, hemodialysis patients, and those with missing data were excluded. The study aimed to calculate the expected partial pressure of carbon dioxide (pCO_2_) values using Winter's formula and simple formula for both arterial and venous blood gas samples and assess potential correlations between them.

Results

The results showed a narrow range for arterial pH values (7.12-7.72), a wider distribution for pCO_2_ values (17.90-81.30 mmHg), and a moderate dispersion for HCO_3_ values (12.80-44.33 mmol/L). Both Winter's and simple formulas were applied to estimate the expected pCO_2_ values, showing strong positive correlations between arterial and venous pH, pCO_2_, and HCO_3_ values. The scatterplot illustrated a very high level of association (Pearson's correlation coefficient, r = 1) between the expected pCO_2_ values derived from both formulas using arterial and venous blood gas samples.

Conclusion

The clinical study demonstrates that estimating expected pCO_2_ values in mixed acid-base disorders can be achieved using a simple and convenient formulation, eliminating the need for arterial blood gas sampling and its associated complications.

## Introduction

In the realm of critical care medicine, the assessment of acid-base balance and gas exchange is of paramount importance for patient management and prognosis. Among the crucial parameters evaluated, the partial pressure of carbon dioxide (pCO_2_) plays a pivotal role in providing insights into respiratory function and the efficiency of pCO_2_ elimination. Whether obtained through arterial blood gas or venous blood gas sampling, the pCO_2_ value serves as a fundamental indicator of a patient's respiratory status and overall acid-base balance.

In intensive care units (ICUs), critically ill patients often require close monitoring and meticulous analysis of blood gases to guide therapeutic interventions and optimize treatment plans. The assessment of pCO_2_, along with other blood gas parameters, forms a cornerstone in understanding the physiological condition of these patients, aiding clinicians in making timely and informed decisions.

Metabolic acidosis-alkalosis, respiratory acidosis-alkalosis, and other less prevalent acid-base disorders can be obscured because of the underlying diseases, necessitating a precise identification of the primary acid-base abnormality for appropriate intervention. For instance, respiratory compensatory mechanisms are activated in mixed acidosis to counterbalance metabolic acidosis [1-3]. It results in an elevation of the respiratory rate, leading to a reduction in blood pCO_2_ levels. Consequently, the compensation process gives rise to an anticipated pCO_2_ value in the context of metabolic acidosis. This expected pCO_2_ value can be determined utilizing either Winter's formula (pCO_2_ = 1.5 × HCO_3_ + 8) or a practical formula (pCO_2_ = 1.2 × HCO_3_ + 11.2) [4]. In instances where the observed pCO_2_ value surpasses the calculated expected pCO_2_ value, the coexistence of respiratory acidosis alongside metabolic acidosis can be identified. Conversely, if the anticipated pCO_2_ value exceeds the measured pCO_2_ value, respiratory alkalosis may be inferred in conjunction with metabolic acidosis. Notwithstanding their practical utility, the retention of these formulas presents challenges due to their inherent complexity. Addressing this concern, a study conducted by Marano et al. [5] proposed an alternative and straightforward approach for determining the anticipated pCO_2_ value in arterial blood gas sampling of hemodialysis patients with metabolic acidosis. The proposed method involves a simple calculation by adding 15 to the HCO_3_ value obtained from venous blood gas (pCO_2_ = HCO_3_ + 15).

Acid-base derangements represent common occurrences in the critical care setting, prompting regular arterial blood gas analyses during patient monitoring. Nonetheless, obtaining arterial blood gas samples entails an interventional procedure involving the insertion of a catheter, thereby exposing patients to potential complications, including bleeding, hematoma, embolism, and aneurysm [6]. Consequently, numerous investigations have centered on exploring the concordance between values derived from arterial and venous blood gas analyses [7-9], given that venous blood gas sampling entails relatively fewer risks and complications compared to its arterial counterpart.

The primary objective of our study is to examine the potential applicability of the "expected (pCO_2_ = HCO_3_ + 15)" value, computed from venous blood gas samples, as an alternative to both Marano's simplified formula and Winter's formula. In this investigation, we conducted a comparative analysis of arterial and venous blood gas samples obtained simultaneously from patients in the intensive care setting.

## Materials and methods

Ethics committee approval (Decision No: 2023-43, Date: March 29, 2023) was secured from the Hitit University Clinical Research Ethics Committee prior to conducting this study. A total of 400 adult patients (50% female, 50% male) aged 18 years and above, who were admitted to the Anesthesia Clinic in the Third Stage Anesthesia and Reanimation Intensive Care Unit at Hitit University Erol Olçok Training and Research Hospital between April 2020 and July 2023, were included.

 Blood gas samples were simultaneously collected from the arterial line and venous catheter. In this database, samples were labeled as “arterial” if the oxyhemoglobin saturation was equal to that displayed by a digital pulse oximeter placed on the hand without vascular access, or if the oxyhemoglobin saturation was larger than or equal to 97%.

 Patients under the age of 18, pregnant women, hemodialysis patients, and patients with missing data were excluded. Among this dataset, we selected 291 blood samples showing HCO_3_ < 24 mmol/L. This cutoff value was chosen to compare our results with those reported in the literature. The patients' median age was 64, with an interquartile range (IQR) of 21. Arterial and venous blood gas analysis results, including pH, HCO_3_, and pCO_2_ values, were recorded for each patient.

 Subsequently, using both Winter's formula (pCO_2_ = 1.5 x HCO_3_ + 8) and simple formula (pCO_2_ = HCO_3_ + 15), the expected pCO_2_ values were calculated for each arterial and venous blood gas sample. The arterial and venous expected pCO_2_ values were gathered for statistical analysis to assess potential correlations.

Statistical analysis

The statistical analysis involved two software programs: IBM SPSS Statistics for Windows, Version 23.0 (Released 2015; IBM Corp., Armonk, New York, United States) and R software (available at https://www.R-project.org/). Categorical variables were expressed as numbers and percentages, while numerical variables were presented as mean ± standard deviation. The normality of numerical variable distributions was assessed using the Kolmogorov-Smirnov test. The Pearson correlation coefficient was employed to evaluate the associations between numerical variables. A scatterplot was generated using the ggplot2 R package (available at https://www.R-project.org/). The level of statistical significance was set at p < 0.05.

## Results

This investigation entailed a comprehensive analysis of arterial and venous blood gas samples obtained from the study participants. Arterial pH values demonstrated a narrow range, with a mean of 7.318 and a standard deviation of 0.12, ranging from 7.12 to 7.72. Similarly, the pCO_2_ values exhibited a wider distribution, with a mean of 39.33 and a standard deviation of 10.917, ranging from 17.90 to 81.30 mmHg. The HCO_3_ values displayed a moderate dispersion, with a mean of 22.334 and a standard deviation of 5.078, varying between 12.80 and 44.33 mmol/L.

For estimating the expected pCO_2_ values, both Winter's and simple formulas were applied. The results obtained from the application of Winter's formula to arterial blood gas samples ranged from 26.80 to 41.75 mmHg, with a mean of 44.82 and a standard deviation of 8.997. Conversely, the expected pCO_2_ values calculated using the simple formula ranged from 25.20 to 54.40 mmHg, with a mean of 42.334 and a standard deviation of 5.92. The analysis of venous blood gas samples showed venous pH values distributed between 7.13 and 7.65, with a mean of 7.262 and a standard deviation of 0.182. The venous pCO_2_ values spanned from 21.91 to 82.50 mmHg, with a mean of 45.856 and a standard deviation of 12.011, while the HCO_3_ values ranged from 12.1 to 42.20 mmol/L, with a mean of 25.919 and a standard deviation of 6.225.

The utilization of Winter's formula to estimate the expected pCO_2_ values from venous blood gas samples yielded results within the range of 26.90-73.10 mmHg, with a mean of 46.91 and a standard deviation of 9.52. On the other hand, the application of the simple formula to venous blood gas samples resulted in expected pCO_2_ values ranging from 27.9 to 59.31 mmHg, with a mean of 41.621 and a standard deviation of 6.034.

The data presented in Table 1 underscore the variability in arterial and venous blood gas parameters and the derived expected pCO_2_ values using Winter's and simple formulas. The results demonstrate the applicability of both formulas to estimate pCO_2_ values from arterial and venous blood gas samples, providing valuable insights for clinical practice and patient management.

**Table 1:** Summary of blood gas analysis of patients (sample size: 400). pCO_2_: partial pressure of carbon dioxide.

Variable	Minimum	Maximum	Mean ± SD	Median (first-third quartile)
Arterial pH	7.12	7.72	7.318 ± 0.12	7.321 (7.278-7.449)
Arterial pCO_2_ values (mmHg)	17.90	81.30	39.33 ± 10.917	38.22 (32.9-42)
Arterial HCO_3_ values (mmol/L)	12.8	44.33	22.334 ± 5.078	22,42 (20.26-28.1)
Expected pCO_2_ values				-
Winter's formula (arterial) (mmHg)	26.80	41.75	44.82 ± 8.997	44.82 (26.80-41.75)
Expected pCO_2_ values				
Simple formula results (arterial) (mmHg)	25.20	54.40	42.334 ± 5.92	42.334 (25.20-54.40)
Venous pH values	7.13	7.65	7.262 ± 0.182	7.389 (7.252-7.425)
Venous pCO_2_ values (mmHg)	21.91	82.50	45.856 ± 12.011	44.9 ( 39-52.1)
Venous HCO_3_ values (mmol/L)	12.1	42.20	25.919 ± 6.225	26.21 (22.2-31.2)
Expected pCO_2_ values				
Winter's formula (venous) (mmHg)	26.9	73.1	46.91 ± 9.52	47.2 (41.52-54.9)
Expected pCO_2_ values				
Simple formula results (venous) (mmHg)	27.9	59.31	41.621 ± 6.034	42.91 (35.9-46.2)

To investigate potential correlations between arterial and venous pH, pCO_2_, HCO_3_, and expected pCO_2_ values computed using Winter's and simple formulas, Pearson correlation coefficient (r) values were calculated. The analysis revealed strong positive correlations between arterial and venous pH values (r = 0.981), pCO_2_ values (r = 0.972), and HCO_3_ values (r = 0.979), as presented in Table 2. Moreover, the expected pCO_2_ values derived from arterial and venous blood gases using both Winter's and simple formulas exhibited a notably high (r = 0.987). Notably, the Pearson correlation coefficient of expected pCO_2_ values calculated with Winter's formula and the simple formula from arterial blood gas samples was 1.0, while the correlation between the expected pCO_2_ values calculated from venous blood gas samples with both formulas also showed a strong correlation coefficient of 1.0 (correlation was significant at the 0.01 level, two-tailed), as summarized in Table 2.

**Table 2:** Correlation between blood gas parameters and formulas for pCO2 estimation. pCO_2_: partial pressure of carbon dioxide.

Variable	Pearson correlation coefficient (r)	p-value
Arterial pH vs. venous pH	0.981	0.001
Arterial pCO_2_ vs. venous pCO_2_ (mmHg)	0.972	0.001
Arterial HCO_3_ vs. venous HCO_3_ (mmol/L)	0.979	0.001
Expected pCO_2_ (Winter's formula) - arterial vs. venous (mmHg)	0.987	0.001
Expected pCO_2_ (Simple formula) - arterial vs. venous (mmHg)	0.987	0.001
Expected pCO_2_ (Winter's formula) - arterial vs. venous (mmHg)	1.0	0.001
Expected pCO_2_ (Simple formula) - arterial vs. venous (mmHg)	1.0	0.001

**Figure 1:**
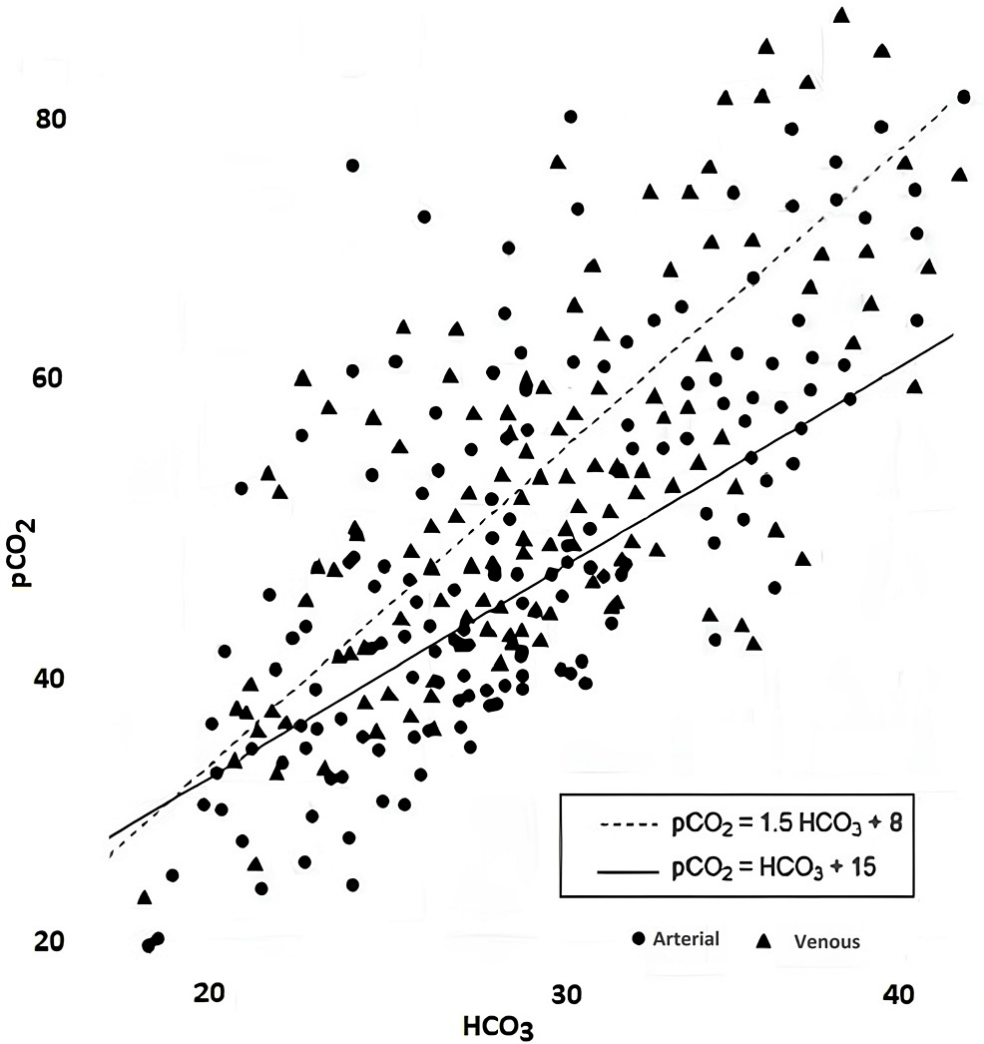
Scatterplot of pCO2 by HCO3. pCO_2_: partial pressure of carbon dioxide.

To visually represent the correlation between Winter's and simple formulas in estimating expected pCO_2_ values, all the aforementioned findings were plotted in a scatterplot (Figure 1). An assessment of arterial and venous pH, pCO_2_, HCO_3_, and expected pCO_2_ values according to Winter's and simple formulas was conducted to investigate potential correlations. The Pearson correlation coefficient (r) was employed as a measure of correlation, with values ranging from 0.90 to 1.00 indicating a very high level of association between the two variables. The statistical significance level was set at p < 0.05.

The scatter plot visually portrays the distribution of arterial and venous pCO_2_ and HCO_3_ values. Within the scatter plot, the expected pCO_2_ values computed using Winter's formula (pCO_2_ = 1.5 × HCO_3_ + 8) are depicted as a dashed line, while the expected pCO_2_ values determined by the simple formula (pCO_2_ = HCO_3_ + 15) are represented by a linear line. Notably, these expected pCO_2_ values were calculated based on data derived from arterial and venous blood gas samples, demonstrating a substantial correlation with each other (Pearson's correlation coefficient, r = 1).

## Discussion

In the realm of intensive care, metabolic acidosis and mixed acidosis are frequent occurrences, necessitating the activation of compensatory mechanisms as a response to diminished HCO_3_ levels and the ensuing acidosis. One crucial compensatory mechanism involves hyperventilation, leading to HCO_3_ retention [10]. Consequently, distinguishing between respiratory and metabolic acidosis in cases of mixed acid-base disorders presents a notable challenge for clinicians. To expedite appropriate interventions and minimize complications, a focused effort has been directed toward pCO_2_ estimation studies, seeking to equip healthcare professionals with rapid and accurate estimations [4,11,12]. Notably, despite extensive research in the field, the establishment of a universally accepted formula for determining the expected pCO_2_ value remains elusive. This limitation underscores the need for further investigations and advancements in the field to enhance clinical decision-making and patient management in critical care settings.

This study aimed to assess the validity and applicability of the Winter formula, which has been extensively investigated in the existing literature, alongside the alternative HCO_3 _+ 15 formula, specifically evaluated in the context of hemodialysis patients, for estimating the expected pCO_2_ value [5,11]. Given the inherent risks associated with arterial blood gas sampling, particularly in critically ill patients within ICUs, several research efforts have been dedicated to addressing these risks and developing a safer and more practical formula for estimating pCO_2_ [4,7,8,13-15].

Our findings shed light on the potential utility of the HCO_3_ + 15 formula as a convenient and straightforward alternative to arterial blood gas sampling, particularly when venous blood gas samples are available. These results align with those reported by Yüksel et al. [4], albeit with the added strength of a larger patient cohort, thereby providing substantial evidence supporting the proposed approach. Furthermore, our study demonstrated a statistically comparable correlation between the simplified HCO_3_ + 15 formula and the more time-consuming Winter's formula, underscoring the promising clinical value of the former in accurately estimating the expected pCO_2_ value.

While these findings hold promise for the clinical management of mixed acid-base disorders in critical care scenarios, it is crucial to acknowledge certain limitations associated with this study. The inclusion of patients from a mixed and heterogeneous population within our university-based unit introduced variability in the collected blood gas samples, potentially influencing the overall generalizability of the results. Additionally, the relatively modest sample size, attributed to the exclusion of patients not requiring arterial blood gas sampling, may have implications for the statistical power of the study.

In light of these limitations, future research endeavors should aim to explore alternative formulas and consider the formation of more homogenous patient groups to strengthen the robustness and reliability of conclusions. Moreover, extending these investigations to encompass a broader range of patient populations would facilitate the comprehensive validation of the proposed HCO_3 _+ 15 formula as a valuable tool in the estimation of expected pCO_2_ values, thereby contributing to improved clinical decision-making and patient care in intensive care settings.

The previous investigation conducted by Marano et al. [5] highlighted a valuable distinction in estimating expected pCO_2_ values based on the HCO_3_ concentration. Specifically, it was suggested that if the HCO_3_ value surpasses 12 mmol/L, the alternative formula (pCO_2_ = HCO_3_ + 15) should be utilized, while Winter's formula (pCO_2_ = 1.5 × HCO_3_ + 8) is deemed more appropriate for cases where HCO_3_ values are below 12 mmol/L. This differentiation is attributed to the Winter formula's limitation in accurately estimating pCO_2_ values in hemodialysis patients with mixed acid-base disorders, where the presence of mean black root error impacts the formula's efficacy. In our current study, hemodialysis patients were deliberately excluded from the analysis, ensuring a more focused examination of critically ill patients without confounding factors related to hemodialysis.

The exclusion of hemodialysis patients enabled a direct comparison of Winter's formula and the alternative HCO_3_ + 15 formula, and no discernible superiority was observed between the two in terms of their performance in estimating pCO_2_ values. Both formulas exhibited comparable accuracy, reinforcing the potential utility of the HCO_3_ + 15 formula as an efficient alternative to Winter's formula in estimating expected pCO_2_ values in mixed acid-base disorder scenarios.

Nonetheless, our study identified a noteworthy value of 12 mmol/L as a significant threshold with implications for future research and clinical applications. While both formulas yielded comparable results in our current patient cohort, the value of 12 mmol/L may hold importance in guiding formula selection in specific clinical contexts or patient populations. Thus, future investigations should aim to explore the clinical significance of this threshold value further and potentially validate its application in identifying the most appropriate formula for estimating expected pCO_2_ values in mixed acid-base disorders.

In summary, our study provides valuable insights into the distinction between Winter's formula and the HCO_3 _+ 15 formula in estimating pCO_2_ values, particularly in mixed acid-base disorder scenarios. The exclusion of hemodialysis patients allowed for a focused assessment of critically ill patients, leading to comparable results between the two formulas. However, the identification of the value of 12 mmol/L as a significant threshold highlights the potential for future research to delve deeper into its clinical implications and applicability in guiding formula selection for accurate pCO_2_ estimation.

Limitations

Our study's outcomes align with previously conducted research, affirming the robustness of the proposed formulations for estimating expected pCO_2_ values. Nonetheless, it is imperative to acknowledge the inherent limitations of our study design. The inclusion of patients from a diverse and heterogeneous population within our university-based unit introduced variability in the collected blood gas samples, limiting the homogeneity of the data. Moreover, the relatively modest sample size could be considered a limitation, primarily due to the exclusion of patients who did not require arterial blood gas sampling. To advance future investigations in the realm of expected pCO_2_ prediction, we advocate exploring alternative formulas that may offer enhanced accuracy and reproducibility. Furthermore, a concerted effort should be made to establish more homogeneous patient groups, facilitating more robust and reliable conclusions. By addressing these considerations, subsequent studies can enhance the applicability and generalizability of the findings, thus contributing to the advancement of clinical practice in the management of mixed acid-base disorders in critical care settings.

## Conclusions

In conclusion, the findings of this clinical study provide valuable evidence that the estimation of expected pCO_2_ values in cases of mixed acid-base disorders can be achieved through the application of a simple and convenient formulation without the necessity of arterial blood gas sampling. This approach offers the advantage of avoiding the potential complications associated with arterial blood gas analysis, thus presenting a promising alternative for clinical use. The study's results underscore the potential significance of implementing such a formulation to expedite the estimation process and enhance patient management in intensive care settings. However, it is essential to acknowledge that further research with larger and more homogenous patient cohorts may be warranted to validate and generalize these findings. Additionally, investigations comparing the proposed simple formula with other existing methods would contribute to a more comprehensive understanding of its clinical utility and accuracy in predicting pCO_2_ values. Nevertheless, the present study offers valuable insights into a potentially practical and efficient approach to addressing mixed acid-base disorders in critical care scenarios.
